# Comparison of surgical trauma between three endoscopic thyroidectomy approaches and open surgery

**DOI:** 10.3389/fonc.2025.1677239

**Published:** 2025-11-20

**Authors:** Jiali Qin, Han Ren, Leilei Li, YaLei Wu, TianMing Xia, Xudong Wang, Liang He

**Affiliations:** 1 Department of Thyroid and Breast Surgery, Affiliated Hospital of Zhengzhou University, Luoyang Central Hospital, Luoyang, Henan, China; 2 Department of Hematology, the First Affiliated Hospital of Henan University of Science and Technology, Luoyang, Henan, China; 3 Department of Head and Neck Oncology, Tianjin Medical University Cancer Institute & Hospital, Tianjin, China; 4 Key Laboratory of Basic and Translational Medicine on Head & Neck Cancer, Tianjin, National Clinical Research Center for Cancer, Tianjin’s Clinical Research Center for Cancer, Tianjin, China

**Keywords:** endoscopic thyroidectomy, thyroid carcinoma, flap dissection area, trauma, thyroid cancer

## Abstract

**Objective:**

This study aims to conduct a comparative analysis of surgical trauma between three endoscopic thyroidectomy approaches (anterior chest, transoral, and unilateral axillary) and conventional open thyroidectomy (COT) in treating unilateral thyroid carcinoma.

**Methods:**

A retrospective analysis was conducted on 200 patients with unilateral differentiated thyroid carcinoma treated at Luoyang Central Hospital affiliated with Zhengzhou University from January 2022 to December 2024. The cohort included 50 patients each: bilateral areolarapproach endoscopic thyroidectomy (BAET), transoral endoscopic thyroidectomy vestibular approach (TOETVA), axillary approach endoscopic thyroidectomy (AAET), and COT. The patients were divided into three groups based on general conditions such as gender, age, and BMI (BAET vs COT; TOETVA vs COT; AAET vs COT).Parameters compared included flap dissection area, perioperative outcomes, postoperative complications, inflammatory markers, postoperative pain (visual analog scale [VAS] score), and cosmetic satisfaction at 3 months;Patients were categorized into normal (BMI < 24 kg/m^2^) and overweight (BMI > 24 kg/m^2^) groups for comparison of flap dissection areas.

**Results:**

The mean age of patients in the three endoscopic thyroidectomy groups was significantly lower than that in the open group (*P* < 0.05). Flap dissection area, operative time, and postoperative drainage volume were significantly higher in the endoscopic groups than in the COT group (*P* < 0.05). No significant differences were observed between the three endoscopic thyroidectomy groups and COT in terms of intraoperative blood loss, days to tube removal, length of hospital stay, number of lymph nodes dissected, incidence of surgical complications, perioperative changes in inflammatory markers (interleukin-6, C-reactive protein, and white blood cells), and pain scores at 1 week postoperatively (*P* > 0.05). However, the TOETVA group exhibited a significantly longer hospital stay and higher VAS pain scores on postoperative day 1 compared with the COT group. No significant differences in these parameters were observed among BAET, AAET, and COT groups (*P* > 0.05). Cosmetic satisfaction was highest in the TOETVA group, followed by the AAET, BAET, and COT groups. Across all surgical groups, patients with normal BMI exhibited significantly smaller flap dissection areas than those in the overweight group (*P* < 0.05).

**Conclusion:**

In appropriately selected patients, total endoscopic thyroidectomy does not significantly increase surgical trauma compared with COT. It offers improved cosmetic outcomes, which supports its favorable clinical applicability.

## 1 Introduction

Most thyroid cancers originate from follicular or parafollicular cells. It is the most common malignancy of the head and neck and ranks among the top ten common malignant tumors (1). Radical thyroidectomy, which may involve unilateral or bilateral thyroid lobes and surrounding lymphoadipose tissue, is one of the commonly used surgeries for thyroid cancer (2). Traditional open thyroidectomy disrupts the anterior cervical anatomy and may impair the function of the anterior cervical region. Additionally, the permanent surgical scar on the neck often causes significant anxiety among patients with heightened aesthetic concerns, especially young patients, owing to the visible disfigurement, which negatively affects their self-image (3). In the past 20 years, advances in endoscopic surgical technology have facilitated the clinical application of total endoscopic thyroidectomy. The integration of 3D visualization systems and high-precision energy equipment has transformed the procedure from being primarily aesthetic-driven to an important part of standardized thyroid surgery.

Transthoracic, transoral, and transaxillary thyroidectomy and other external cervical approaches have gained popularity in clinical practice because of their concealed incisions, which leave no visible neck scars. These techniques preserve the anatomy and function of the anterior cervical region and have little impact on patients’ postoperative life and work, thereby offering excellent psychological benefits (4). Additionally, these approaches confer physiological benefits, including small incisions, rapid healing, reduced intraoperative blood loss, and reduced postoperative pain. However, some experts believe that full endoscopic thyroidectomy is not minimally invasive in the traditional sense because of the larger extent of flap dissection compared with open surgery (5). Currently, evidence-based international research comparing systemic trauma associated with full endoscopic and open surgery is lacking.

Existing studies often focus on isolated parameters and are limited by the lack of standardized systems for quantifying surgical trauma. In this study, we conducted a more comprehensive and objective evaluation of the mainstream clinical approach of full endoscopic thyroid surgery and open surgery. The analysis focused on differences in anatomical trauma, inflammatory stress, metabolic disturbances, and other differential indicators to provide an evidence-based basis for clinical decision-making and the selection of appropriate surgical methods for patients with clinically differentiated thyroid cancer.

## 2 Methods

### 2.1 General information

A total of 200 patients with unilateral differentiated thyroid cancer who were treated at our hospital between January 2022 and December 2024 were enrolled and divided into 4 groups based on the surgical approach: (1) conventional open thyroidectomy (COT); (2) bilateral areolarapproach endoscopic thyroidectomy (BAET); (3) transoral endoscopic thyroidectomy vestibular approach (TOETVA); (4) axillary approach endoscopic thyroidectomy (AAET). Patients were further grouped by gender, body mass index (BMI), and case frequency as follows: (1) BAET group (n = 50) and COT group (n = 50), (2) TOETVA group (n = 50) and COT group (n = 50), and (3) AAET group (n = 50) and COT group (n = 50).The surgeries described above were performed by surgeons from different operative teams, with each surgeon having accumulated over 300 cases of experience in the specific surgical procedure they conducted.

The inclusion criteria included (1) unilateral thyroid lobectomy with ipsilateral central lymph node dissection; (2) pathologically confirmed differentiated thyroid carcinoma without central lymph node metastasis or with ≤ 5 metastatic lymph nodes; (3) maximum tumor diameter < 2 cm, without extensive local invasion or adhesion; (4) age ≥ 18 years; (5) no previous history of thyroid surgery.

The exclusion criteria included (1) maximum tumor diameter > 2 cm, invasion of adjacent structures (including trachea, esophagus, recurrent laryngeal nerve, and blood vessels), fused or cystic metastatic lymph nodes, mediastinal invasion, or extensive metastasis; (2) coagulation disorders and severe infectious diseases that contraindicate surgery; (3) presence of other malignant tumors; (4) presence of systemic autoimmune diseases. All research procedures in this study were reviewed and approved by the ethics committee, and informed consent was obtained from all participants (approval batch number: LWLL-2023-10-11-01).

### 2.2 Surgical method

**Figure 1:**
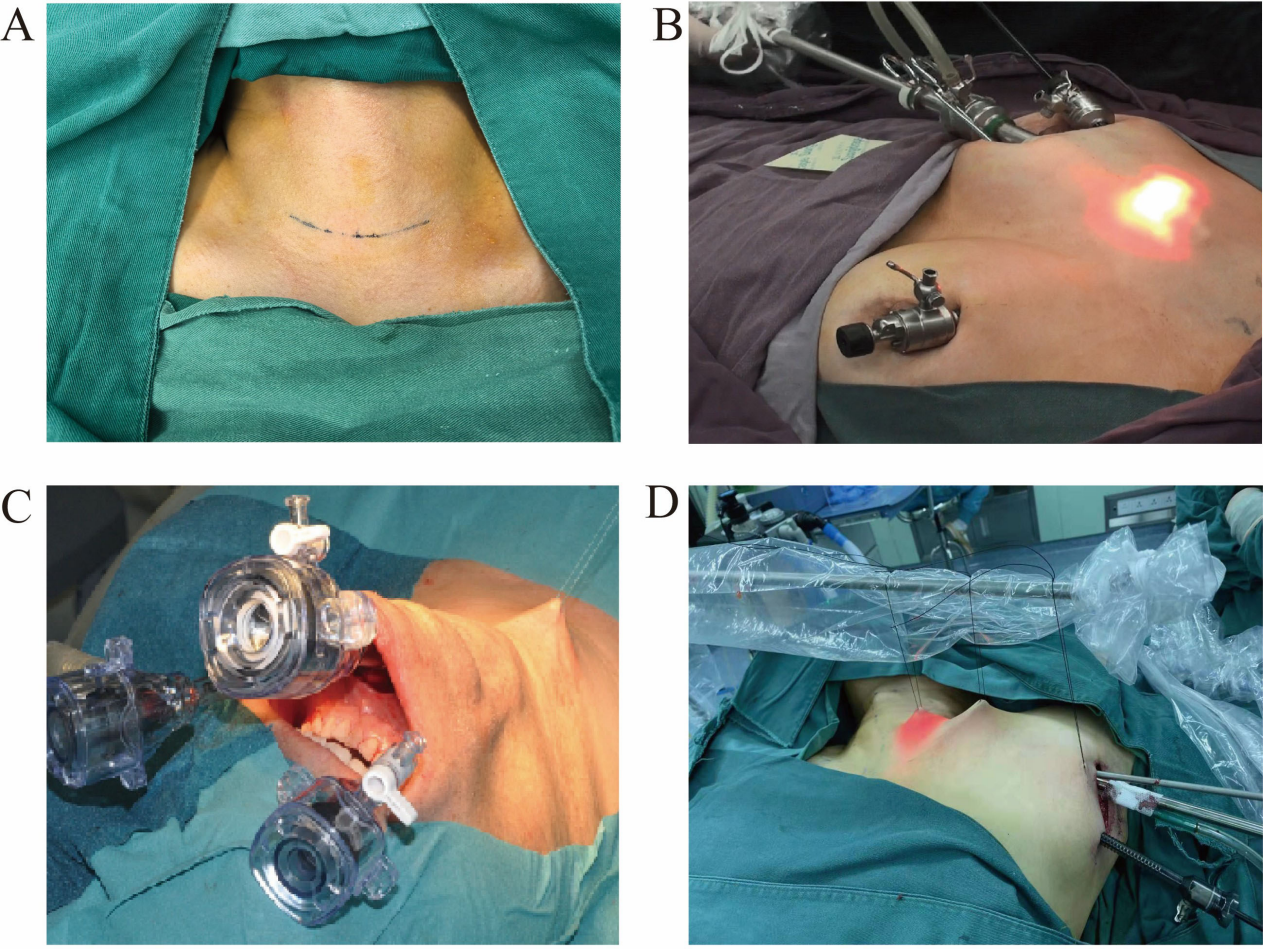
Four surgical approaches **(A)** Conventional open thyroidectomy. **(B)** Bilateral areolarapproach endoscopic thyroidectomy. **(C)** Transoral endoscopic thyroidectomy vestibular approach. **(D)** Axillary approach endoscopic thyroidectomy.

**Figure 2:**
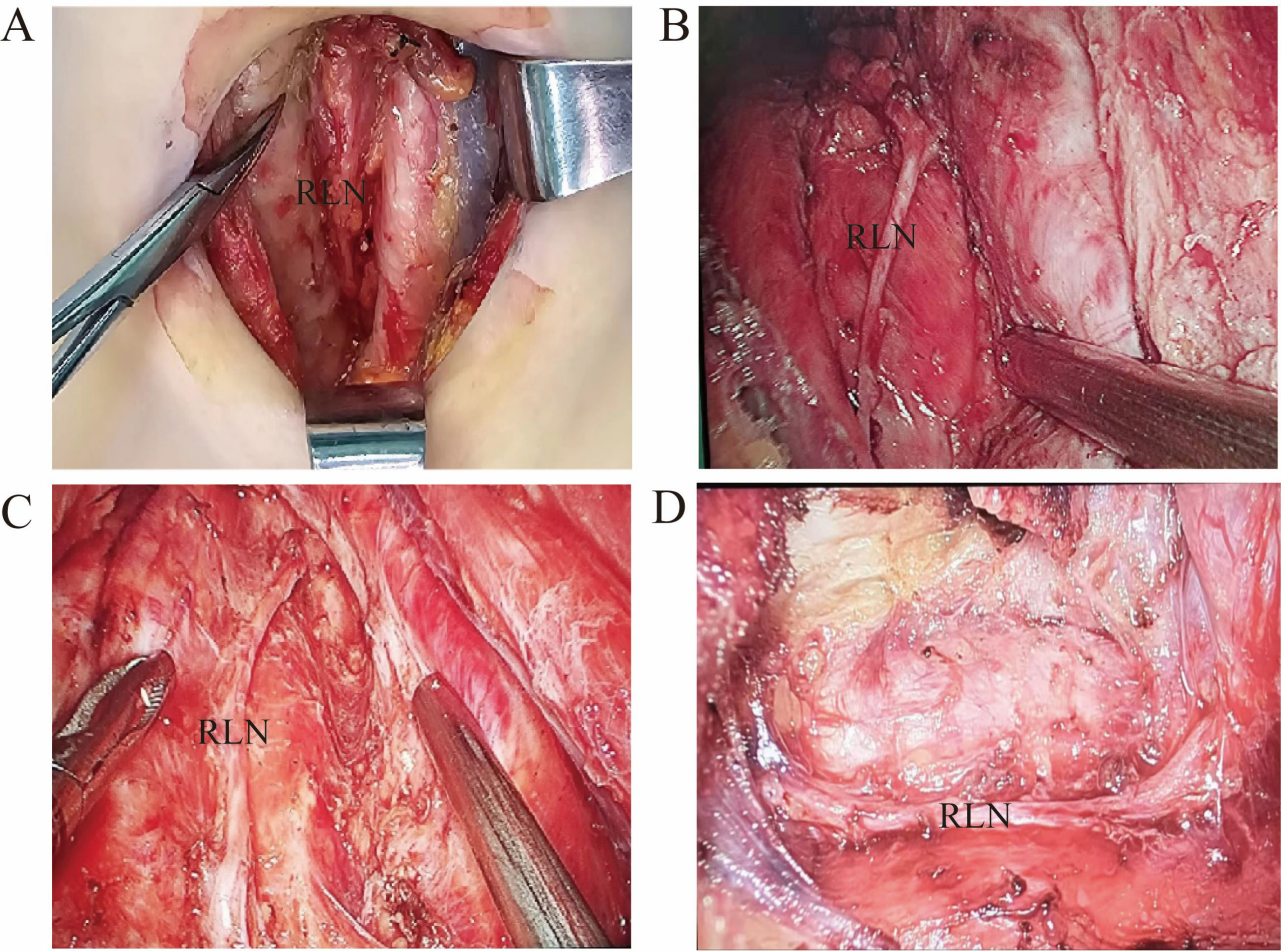
Four intraoperative photos of the surgeries **(A)** Conventional open thyroidectomy. **(B)** Transoral endoscopic thyroidectomy vestibular approach. **(C)** Transoral endoscopic thyroidectomy. **(D)** Axillary approach endoscopic thyroidectomy.

Conventional open thyroidectomy (**Figures 1A**, **2A**): The patient underwent endotracheal intubation under general anesthesia in the supine position. After routine disinfection and draping, a 5 cm low-collar curved incision was made along the cervical skin lines approximately 2 cm above the suprasternal notch. The surgical procedure proceeded with layer-by-layer dissection through the skin, subcutaneous fat, and platysma muscle. Following the elevation of the skin flaps, the midline raphe was incised longitudinally to access the thyroid gland. After opening the outer capsule, the perithyroidal vessels were carefully ligated. Meticulous dissection was performed to identify and preserve the recurrent laryngeal nerve, parathyroid glands, and common carotid artery. Lobectomy was completed with en bloc resection, followed by prophylactic ipsilateral central compartment lymph node dissection.

Bilateral areolarapproach endoscopic thyroidectomy (**Figures 1B**, **2B**): The patient underwent tracheal intubation under general anesthesia in the supine position. After routine disinfection and draping, a 1.5 cm long longitudinal incision was made at the 2–3 o’clock position of the right areola. A separating rod was used to dissect the subcutaneous tissue to the bilateral clavicles, and a 1 cm diameter trocar was inserted. Additional 0.5 cm incisions were made at the 11 o’clock position of the left areola and the 12 o’clock position of the right areola, and 5 mm trocars were inserted. The skin flap was separated from the suprasternal fossa to the thyroid cartilage position. The pneumoperitoneum pressure was set at 6 mmHg, with a flow rate of 40 L/min. An ultrasonic scalpel was used to expose the thyroid tissue, coagulate surrounding blood vessels, incise the outer membrane, and expose and preserve the recurrent laryngeal nerve, parathyroid glands, and common carotid artery. The thyroid lobe was then completely removed, followed by prophylactic dissection of lymphoid fat tissue in the ipsilateral central region.

Transoral endoscopic thyroidectomy (**Figures 1C**, **2C**): After orotracheal intubation under general anesthesia, the endotracheal tube was secured. The patient was placed in the supine position with slight neck extension and shoulder elevation, and a towel was routinely spread. The oral cavity was rinsed with diluted iodophor. A 2 cm transverse incision was made approximately 1 cm away from the gingival base in front of the lower lip frenulum, extending diagonally toward the inner surface of the mandible. Two additional 5 mm longitudinal incisions were made in the buccal mucosa at the root of the bilateral first premolars. Blunt dissection was performed to avoid injury to the mental nerve. The flap was separated using a combination of blunt and sharp dissection, following the inner surface of the mandible close to the periosteum, and extended toward the submental region. The recurrent laryngeal nerve was dissected, and the parathyroid gland was preserved. The unilateral thyroid gland was completely resected, followed by ipsilateral central lymph node removal. The surgical cavity was flushed, and after hemostasis, the incision was closed in layers. A drainage tube was placed in the anterior neck, and the incision was sutured.

Axillary endoscopic thyroidectomy (**Figures 1D**, **2D**): The patient was placed in the supine position, with the neck slightly tilted to the unaffected side, allowing full exposure of the affected axilla. Following the use of general anesthesia and routine sterilization of the surgical area, a 4 cm incision was made along the first skinfold of the axilla for the insertion of the observation endoscope and operating instruments. Approximately 3.0 cm below this incision, another 0.5 cm incision was made to serve as an operating hole. The approach involved the separation of the sternocleidomastoid space, scapulohyoid muscle, and internal jugular vein to expose the thyroid gland. The recurrent laryngeal nerve was exposed, the superior pole of the thyroid gland was managed, and the parathyroid gland was preserved. The thyroid lobe on the affected side was excised, and the specimen was removed. The common carotid artery sheath was separated, and the recurrent laryngeal nerve was dissociated throughout the procedure. Central lymph nodes were excised, and the resected specimen was removed. The wound was irrigated, a drainage tube was placed, and the incisions were sutured.

### 2.3 Observed metrics

#### 2.3.1 Surgical conditions

The following metrics were observed: operation time, intraoperative blood loss, postoperative drainage volume, timing of drainage tube removal, length of hospital stay, and number of lymph nodes dissected. The duration of the operation was from the beginning of the skin incision to the end of the suture. Postoperative drainage volume was calculated as the total volume of fluid drained daily from the day of surgery to the day of extubation.

#### 2.3.2 Postoperative complications

The observed complications included hemorrhagic hematoma, postoperative infection, parathyroid gland injury, and recurrent laryngeal nerve injury.

#### 2.3.3 Inflammatory response

A 3 mL sample of fasting venous blood was collected from the elbow in the morning, 1 day before surgery and 1 day after surgery. The sample was centrifuged at 3000 r/min for 15 min, and the serum was separated and stored at −80°C. Serum levels of interleukin-6 (IL-6) and C-reactive protein (CRP) were measured using enzyme-linked immunosorbent assay kits (eBioscience, USA). White blood cell (WBC) counts were determined using the XT-5000i automatic hematology analyzer (Sysmax, Japan). All procedures were performed in strict accordance with the manufacturer’s instructions and instrument operating guidelines.

#### 2.3.4 Measurement and calculation of the flap dissection area

Before surgery, the skin surface corresponding to the planned flap dissection area was marked. endoscopic surgery: Before the end of the procedure, the endoscopic light source was used to illuminate the subcutaneous area. The boundary of the surgical area was identified under the skin with dissecting forceps, marked externally with methylene blue applied via a cotton swab, and then the area was measured and calculated. Open surgery: Before the end of the operation, the boundary of the surgical area was marked by extending surgical forceps into the flap. The boundary was then traced with methylene blue using a cotton swab, and the area was measured and calculated. After obtaining the parameters from the example diagram, keeping the camera position, angle, patient’s position, and camera settings fixed allowed these results to be directly applied to calculate the area in subsequent photographs.

The Multi Distance Meter in 3ds Max was used to measure distances and surface areas of the surgical models. The relevant scene or model was opened in 3ds Max, and the Multi Distance Meter was activated via the plug-in menu. The “Measure” function was selected, and two points within the viewport were designated for linear measurement. The results were displayed in the measurement window, with connection lines and labeled values automatically rendered on the line segment. To measure plane areas, the built-in “Measure” tools in 3ds Max were employed. The area option was selected in the tool panel, and the target surface was selected within the viewport. The software automatically computed and displayed the area of the selected region (6).

#### 2.3.5 Postoperative pain assessment

Postoperative pain was assessed using a visual analog scale (VAS), with a score of 0 indicating no pain, 1–3 indicating mild pain, 4–6 indicating strong but tolerable pain, and 7–10 indicating severe and unbearable pain. Pain evaluations were conducted during follow-up visits either at outpatient clinics or via telephone calls (7).

#### 2.3.6 Aesthetic satisfaction

After 3 months of follow-up, incision aesthetics were evaluated based on four criteria: subjective satisfaction, skin color, texture and elasticity, and the appearance of the surgical scar. Each criterion was scored from 1 to 4, with total scores ranging from 4 to 16. A score of 12 to 16 was rated as excellent, 8 to 11 as good, and 4 to 7 as poor.Aesthetic satisfaction was calculated as follows: (excellent cases +good cases)/total number of cases × 100% (8).

### 2.4 Statistical analysis

Statistical Package for the Social Sciences software (version 26.0) was used for data analysis, and *P* < 0.05 was considered statistically significant. The general clinical data of the patients included gender, age, and BMI, and the relevant data of the operation included the area of flap separation, operation time, surgical blood loss, postoperative drainage, postoperative pain, and aesthetic satisfaction. Thyroid function and thyroid ultrasound were re-examined 3 months after surgery and every 6 months thereafter, and follow-up was conducted until 24 months after surgery.

## 3 Results

### 3.1 Comparison of general data between each endoscopic surgery group and the open surgery group

No significant differences were observed in gender, BMI, tumor diameter, and central lymph node metastasis among the groups, as determined by the chi-square test, indicating that the groups were comparable (**Table 1**). Because the patients in the endoscopic group were generally young, age differences between the three groups and the open group were analyzed using the *t*-test. Results indicated that the mean age of patients in the three groups was significantly lower than that of the open group (*P* < 0.05; **Table 2**).

**Table 1:** Comparison of general conditions between the endoscopic surgery group and the conventional open thyroidectomy group.

Variables	BAET (n=50)	TOETVA (n=50)	AAET (n=50)	COT (n=50)
Sex				
Male	14	10	9	15
Female	36	40	41	35
χ2	0.05	1.33	1.97	–
P	0.83	0.25	0.16	–
Obesity (BMI≥24)				
Yes	22	15	16	24
No	28	35	34	26
χ2	0.16	3.41	2.67	–
P	0.69	0.07	0.10	–
Tumor diameter				
≥1cm	20	21	16	23
<1cm	30	29	34	27
χ2	0.37	0.16	2.06	–
P	0.55	0.69	0.15	–
Central lymph node				
transfer	23	22	19	21
not transferred	27	28	31	29
χ2	0.16	0.04	0.17	–
P	0.69	0.84	0.68	–

**Table 2:** Comparison of patient age between the endoscopic surgery group and the conventional open thyroidectomy group( ± S, *n* = 50).

Group	Age	*t*	*P*
BAET	40.58 ± 9.11	-4.83	0.00
TOETVA	38.66 ± 8.19	-5.99	0.00
AAET	38.80 ± 9.35	-5.63	0.00
COT	50.54 ± 11.38	–	–

### 3.2 Comparison of surgical data between the endoscopic surgery group and the open surgery group

The endoscopic surgery group exhibited a significantly greater total flap separation area, longer operation duration, and higher postoperative drainage volume compared with the open surgery group (*P* < 0.05).the cervical flap separation area in the BAET group was significantly smaller than that in the open surgery group (*P* < 0.05). The TOETVA group exhibited a significantly extended hospital stay compared with the open surgery group (*P* < 0.05).The differences in intraoperative blood loss, timing of drainage tube removal and number of lymph node dissections between the endoscopic surgery group and the traditional open thyroidectomy group were statistically non-significant (*P* > 0.05).The differences in hospital stay between the BAET group, the AAET group and the traditional open thyroidectomy group were statistically non-significant (*P* > 0.05)(**Table 3**).

**Table 3:** Comparison of surgical conditions between the endoscopic surgery group and the conventional open thyroidectomy group( ± S, *n* = 50).

Group	Total flap separation area (cm)	Cervical segment area of flap separation (cm)	Operation time (min)	Intraoperative blood loss (mL)
BAET	120.76 ± 8.29	71.42 ± 8.93	90.60 ± 10.18	10.50 ± 5.74
*t*	29.48	-3.63	18.34	0.74
*P*	0	0	0	0.46
TOETVA	113.71 ± 9.77	–	108.70 ± 10.44	9.40 ± 4.48
*t*	22.18	–	26.73	-0.31
*P*	0	–	0	0.76
AAET	175.70 ± 10.55	–	77.80 ± 9.10	11.00 ± 5.25
*t*	56.51	–	12.82	1.26
*P*	0	–	0	0.21
COT	77.06 ± 6.41	77.06 ± 6.41	52.48 ± 10.60	9.70 ± 5.09
Group	Postoperative drainage volume (mL)	Timing of drainage tube removal (days)	Length of stay (days)	Number of lymph nodes dissected (pieces)
BAET	139.32 ± 31.12	3.10 ± 0.54	7.74 ± 1.14	6.06 ± 1.50
*t*	5.65	1.18	1.12	-1.59
*P*	0	0.24	0.27	0.12
TOETVA	135.06 ± 52.01	3.16 ± 0.51	8.42 ± 1.13	6.06 ± 1.46
*t*	3.76	1.83	4.33	-1.62
*P*	0	0.07	0	0.11
AAET	156.96 ± 41.71	3.20 ± 0.64	7.88 ± 1.22	6.14 ± 1.58
*t*	7.15	1.96	1.7	-1.27
*P*	0	0.54	0.09	0.21
COT	101.56 ± 35.54	2.98 ± 0.47	7.50 ± 0.99	6.50 ± 1.25

### 3.3 Effects of different body mass indices on flap separation area in each group

Using a BMI of 24 kg/m^2^ as the threshold, patients were categorized into normal and overweight groups, and the flap separation area was compared. The flap separation area in patients with normal BMI was significantly smaller than that of patients who were overweight in the BAET, TOETVA, AAET, and COT groups (*P* < 0.05) (**Table 4**).

**Table 4:** Comparison of flap separation area between the normal group and the overweight group.

group	BAET (cm^2^)	TOETVA (cm^2^)	AAET (cm^2^)	COT (cm^2^)
normal group	118.13 ± 8.40	111.33 ± 10.02	173.61 ± 10.95	74.54 ± 4.88
overweight group	124.12 ± 6.99	119.27 ± 6.54	180.13 ± 8.32	79.79 ± 6.83
*t*	-2.693	-2.812	-2.105	-3.148
*P*	0.01	0.007	0.041	0.003

### 3.4 Comparison of surgical complications between each endoscopic surgery group and the open surgery group

No cases of postoperative subcutaneous hematoma or infection were observed in any of the groups, including the BAET, TOETVA, AAET, and COT groups, indicating no significant differences in these complications among the groups. Because unilateral thyroid surgery rarely results in parathyroid gland damage, only recurrent laryngeal nerve injury was compared. No permanent recurrent laryngeal nerve injury occurred in any group. One case of transient recurrent laryngeal nerve injury was reported in the COT group, with vocal function returning to normal approximately 50 days postoperatively. In the BAET group, two cases of transient recurrent laryngeal nerve injury were recorded, with recovery of normal vocal function around 50 days postoperatively. In the TOETVA group, three cases of transient recurrent laryngeal nerve injury were recorded; vocal recovery occurred in one case after 40 days and in two cases after approximately 45 days. In the AAET group, two cases of transient recurrent laryngeal nerve injury were recorded, with recovery of normal vocal function around 45 days postoperatively. No permanent recurrent laryngeal nerve injury occurred in any group, and no significant differences were observed between the groups (**Table 5**).

**Table 5:** Comparison of postoperative recurrent laryngeal nerve injury between the endoscopic surgery group and the conventional open thyroidectomy group.

Group	Recurrent laryngeal nerve injury	
	Yes	No
BAET	2	48
TOETVA	3	47
AAET	2	48
COT	1	47

### 3.5 Comparison of inflammatory factor levels between the endoscopic surgery group and the open surgery group

The serum levels of IL-6, CRP, and WBC were compared between groups using the *t*-test. No significant differences were observed in the preoperative levels (*P* > 0.05). Similarly, no significant differences were observed in the postoperative levels on the first day after surgery (*P* > 0.05) (**Table 6**).

**Table 6:** Comparing the serum levels of IL-6, CRP and white blood cells between the endoscopic surgery group and the conventional open thyroidectomy group.( ± S, *n* = 50).

Group	WBC (×10^9^/L)		IL-6 (pg/mL)		CRP (pg/mL)	
	Preoperative Day 1	Postoperative Day 1	Preoperative Day 1	Postoperative Day 1	Preoperative Day 1	Postoperative Day 1
BAET	5.66 ± 1.20	10.28 ± 2.40	1.83 ± 1.13	4.24 ± 3.44	1.19 ± 1.51	3.85 ± 3.83
t	-0.628	-0.561	-1.064	-1.787	-1.186	-1.647
P	0.532	0.576	0.29	0.077	0.239	0.103
TOETVA	5.31 ± 1.17	9.86 ± 2.61	2.01 ± 1.12	4.21 ± 3.61	1.10 ± 1.08	5.09 ± 4.14
t	-1.961	-1.3	-0.214	-1.774	-1.622	0.077
P	0.053	0.197	0.83	0.079	0.108	0.939
AAET	5.43 ± 1.01	11.49 ± 3.30	1.95 ± 0.94	5.97 ± 4.69	1.37 ± 1.76	6.18 ± 3.15
t	-1.595	1.461	-0.562	0.726	-0.593	1.773
P	0.114	0.147	0.575	0.47	0.555	0.079
COT	5.82 ± 1.43	10.58 ± 2.93	2.06 ± 0.96	5.39 ± 3.03	1.58 ± 1.78	5.03 ± 3.34

### 3.6 Comparison of postoperative pain among the endoscopic surgery groups

Follow-up was conducted via telephone, work WeChat, and outpatient visits, and all patients were followed up. On day 1 after surgery, the VAS pain score in the TOETVA group was significantly higher than that in the open surgery group (*P* < 0.05). No significant differences in VAS pain scores were observed among the BAET, AAET, and open surgery groups (*P* > 0.05). At 1 week after surgery, no significant differences in VAS pain scores were observed among the groups (*P* > 0.05) (**Table 7**).

**Table 7:** Comparison of postoperative pain levels between the endoscopic surgery group and the conventional open thyroidectomy group .( ± S, *n* = 50).

Group	Postoperative day 1 VAS	Postoperative week 1 VAS
BAET	3.12 ± 1.04	1.26 ± 0.69
t	-1.701	0.305
P	0.092	0.761
TOETVA	4.64 ± 1.14	1.44 ± 0.81
t	5.241	1.526
P	0.000	0.130
AAET	3.12 ± 1.02	1.18 ± 0.69
t	-1.717	-0.306
P	0.089	0.761
COT	3.48 ± 1.07	1.22 ± 0.62

### 3.7 Comparative analysis of aesthetic satisfaction at 3 months after surgery in the endoscopic surgery groups

Follow-up was conducted via telephone, work WeChat, and outpatient visits, and all patients were followed up. The aesthetic satisfaction rates were 96% in the TOETVA group, 84% in the AAET group, 82% in the BAET group, and 60% in the COT group (**Table 8**).

**Table 8:** Comparative analysis of cosmetic outcomes among surgical approaches.(*n* = 50).

Group	Excellent	Good	Poor	Aesthetic outcome
BAET group	32	9	9	82%
TOETVA group	45	3	2	96%
AAET group	30	12	8	84%
COT Group	20	10	20	60%

## 4 Discussion

In the last two decades, the age-standardized incidence of thyroid cancer has been increasing worldwide (9).Surgery is the most effective treatment for thyroid cancer. Traditional open surgery, which is the standard surgical approach, has demonstrated a 10-year survival rate exceeding 90%, and its efficacy has been widely recognized in clinical practice (10, 11). However, traditional open surgery often results in visible scarring and reduced cutaneous tactile function, which is poorly accepted by many patients, especially young women (3). With advances in minimally invasive techniques, the first endoscopic parathyroidectomy was reported by Gagnert et al. in 1996 (12), followed by the first endoscopic thyroid lobectomy performed by Huscher et al. in 1997 (13). Since then, endoscopic thyroid surgery has developed rapidly owing to its minimally invasive nature, enhanced surgical visualization, and safety. Various approaches to endoscopic thyroid surgery have emerged, including transthoracic, transaxillary, subclavian, and transoral (14). The most commonly used in clinical practice are transthoracic, transaxillary, and transoral approaches. endoscopic thyroid surgery has minimal impact on patients’ postoperative life and work owing to its concealed incision and absence of visible neck scars, thereby offering excellent psychological and cosmetic benefits. However, some believe that fully endoscopic thyroidectomy is not minimally invasive in the traditional sense because the extent of flap separation is greater than in open surgery (5).

Thyroid cancer is more prevalent among females, with a male-to-female ratio of 1:3. Increasing evidence suggests that estrogen plays an important role in the pathogenesis of thyroid tumors, which may explain the higher incidence among women (15). Additionally, the incidence of thyroid cancer has shifted toward younger age groups recently (16). Endoscopic thyroid surgery is more acceptable among young women because of its favorable cosmetic results. In this study, patients who underwent endoscopic thyroidectomy across all approaches were younger than those who underwent open surgery. This suggests that endoscopic surgery is more acceptable in younger populations with greater cosmetic concerns.

Body mass index is a widely accepted international metric for assessing obesity. It is often used to evaluate health and nutritional status. Obesity was previously considered a relative contraindication for endoscopic thyroid surgery owing to challenges such as unclear anatomical planes and increased surgical complexity (17). However, advances in technology and surgical equipment have broadened the indications for endoscopic surgery (18). In this study, patients with higher BMI consistently exhibited larger flap separation areas compared with those with lower BMI, regardless of whether the surgery was endoscopic or open. Currently, the controversy over whether endoscopic thyroid surgery is minimally invasive is that the flap separation area is too large. To accurately compare the flap separation area across different approaches, computer modeling was conducted. Postoperative demarcation of flap boundaries was performed using fixed-angle photography, and flap separation areas were calculated in 3ds Max using Multi Distance Meter software. Results revealed that the flap separation area in all endoscopic approaches was larger than in open surgery. However, in the transthoracic endoscopic group, the cervical flap separation area was smaller than in open surgery. The difference was mainly due to the separation range of the subcutaneous chest tunnel formed during trocar insertion. Unlike cervical flap separation, this tunnel can self-collapse following trocar removal, implying less actual trauma than its surface area suggests. In addition, all endoscopic approaches were performed on the deep surface of the platysma muscle, thus preserving the continuity of the neck skin, subcutaneous tissue, and platysma muscle. As a result, patients experienced significantly reduced postoperative neck pain, tightness, and foreign body sensation.

Compared with open surgery, endoscopic thyroid surgery requires a slightly longer operative time owing to longer preparation time and the need to construct a working cavity (19). This study confirmed that the operative durations for the anterior thoracic, transoral, and transaxillary approaches were longer than that of open surgery. However, operative time is not a primary indicator of the size of the wound, as it is influenced by the surgeon’s technical proficiency and the surgical equipment used. endoscopic thyroid surgery may result in increased postoperative drainage owing to the larger flap separation area compared with open surgery. In this study, all three endoscopic approaches resulted in higher drainage volumes than open surgery. With continuous improvement in surgical techniques and surgical instruments, this difference is expected to gradually diminish. The length of hospital stay in the transoral approach group was 1 day longer than in the open surgery group, while no significant differences were observed between the other two groups. This extended stay may be because transoral thyroid surgery requires preoperative antibiotic preparation. In addition, patients in the transoral endoscopy group experienced relatively greater damage to the platysma muscle. Because the surgical area was The nerve density, postoperative pain was more pronounced than in the open surgery group. However, the transoral approach provided significantly better postoperative aesthetics than the other three groups, as the incision was made within the oral cavity, leaving no visible scar on the body surface. The absence of external scarring contributed to improved cosmetic satisfaction. Furthermore, the postoperative aesthetic outcome of the axillary approach is superior to that of the thoracic approach. We hypothesize that this observation may be attributed to two primary factors. First, the majority of patients undergoing this surgery are young women, for whom the breast—functioning as a sexual organ—carries special significance. Many of these patients express notable concern regarding areolar scars, a common sequela of the thoracic approach. In contrast, although the axillary approach may result in a relatively larger scar, its location in a naturally concealed axillary region still confers excellent aesthetic results. Second, the selection criteria of the surgical samples in the present study may also have contributed to the observed discrepancy in aesthetic outcomes.

In this study, no significant differences were observed between the three endoscopic thyroid surgery approaches and open surgery in terms of intraoperative blood loss, number of days of extubation, recurrent laryngeal nerve injury, subcutaneous hematoma, incision infection, pain at 1 week after surgery, and the number of lymph node dissections. Papillary thyroid carcinoma exhibits a relatively high rate of central compartment lymph node metastasis, and the number of dissected lymph nodes serves as one of the key evaluative indicators. In the present study, no significant difference was observed in this metric among the three endoscopic surgery groups and the traditional open surgery group. This finding differs slightly from those of previous studies (3).Notably, compared with other approaches, the transoral vestibular approach facilitates central lymph node dissection from a superior-to-inferior angle, which allows for adequate exposure of blind zones such as the clavicular and sternal regions and enables more thorough clearance of central neck lymph nodes. Consequently, this approach typically yields a greater number of dissected lymph nodes than alternative techniques. However, authoritative guidelines (10) clearly delineate the boundaries for central compartment lymph node dissection: the inferior boundary is defined as the upper edge of the subclavian artery, the superior boundary as the level of the thyroid cartilage, and the lateral boundaries as the medial margins of the common carotid arteries, encompassing the anterior tracheal region. For the right tracheoesophageal groove, particular attention should be paid to lympho-adipose tissue deep to the level of the recurrent laryngeal nerve. In the current study, the scope of central compartment lymph node dissection in all three endoscopic surgery groups was strictly performed in accordance with these defined boundaries. Additionally, the number of dissected lymph nodes may also be partially influenced by pathological processing protocols.

With the rapid advancement of endoscopic thyroid surgery techniques, this approach has gained widespread clinical adoption. Although endoscopic surgery offers numerous advantages over conventional open procedures, concerns remain regarding whether its longer operative duration and more extensive flap dissection may cause greater physiological trauma (20). Current evidence indicates that the degree of surgical trauma correlates positively with the body’s stress response. Surgical injury triggers a cascade of neuroendocrine changes that promote the release and expression of stress-related inflammatory mediators, among which IL-6, CRP, and WBC are representative biomarkers (21). Interleukin-6, a pleiotropic cytokine produced by Th2 cells, fibroblasts, and macrophages, increases following surgical trauma and decreases as healing progresses (22). C-reactive protein, an acute-phase protein closely associated with stress response, is rapidly synthesized and released upon tissue injury, with serum levels reflecting the severity of trauma (23). White blood cell count serves as another crucial indicator for evaluating surgical stress and trauma severity (24). In this study, differences observed in serum IL-6, CRP, and WBC levels between endoscopic and open surgery groups on postoperative day 1 were statistically non-significant. These findings suggest that the three endoscopic approaches do not impose greater surgical trauma compared with conventional thyroidectomy. Furthermore, owing to their minimally invasive nature and smaller, well-concealed incisions, endoscopic thyroid surgeries offer superior cosmetic outcomes and facilitate faster postoperative recovery, enabling patients to resume normal social and family life more quickly. These advantages support their broader clinical adoption.

In summary, although various endoscopic thyroidectomy approaches involve a larger flap dissection area, slightly longer operative time, and somewhat greater postoperative drainage volume compared with open surgery, they do not significantly increase patient trauma in other aspects. For appropriately selected patients, endoscopic thyroidectomy can achieve therapeutic outcomes equivalent to those of open surgery while offering advantages in terms of reduced physiological and psychological trauma. Therefore, it holds favorable clinical value and should be regarded as a truly minimally invasive surgical approach.

## 5 Limitations

A limitation of the present study is that all enrolled patients had unilateral thyroid cancer. Owing to the influence of other disease-related factors, selection bias was unavoidable. Notably, this study explicitly standardized the surgical protocol for each group to unilateral intervention only, ensuring comparability across groups. However, the study is constrained by a small sample size and a short follow-up duration, highlighting the need for further analyses with larger sample sizes in future research.

Subsequently, we plan to design a multi-center, large-sample, prospective randomized controlled study to compare endoscopic thyroidectomy with traditional open surgery. This future study aims to further validate the safety, oncological radicality, minimally invasive properties, and aesthetic outcomes of endoscopic thyroidectomy.

## Limitations

The limitations of this study should be acknowledged. First, our study was retrospective, so inherent biases associated with this design might have reduced the statistical power. Second, the clinicopathological parameters were disproportionally distributed in the patients enrolled in our study, which resulted in a large error in the relevant statistical analysis.

## Data Availability

The original contributions presented in the study are included in the article/supplementary material. Further inquiries can be directed to the corresponding authors.
